# PVP-Regulated Self-Assembly of High-Strength Micrometer-Scale Al/CuO/AP Energetic Microspheres with Enhanced Reactivity

**DOI:** 10.3390/polym17141994

**Published:** 2025-07-21

**Authors:** Xuyang Wu, Hongbao Wang, Chenglong Jiao, Benbo Zhao, Shixiong Sun, Yunjun Luo

**Affiliations:** 1 School of Environmental and Safety Engineering, North University of China, Taiyuan 030051, China; wuxuyang2003@163.com (X.W.); 17200585545@163.com (H.W.); 2 School of Chemistry and Chemical Engineering, North University of China, Taiyuan 030051, China; jiaocl2000@163.com (C.J.); zhaobenbo@163.com (B.Z.); 3 Dezhou Industrial Technology Research Institute, North University of China, Dezhou 253034, China; 4 School of Materials Science and Technology, Beijing Institute of Technology, Beijing 100081, China

**Keywords:** self-assembly, micrometer-sized Al/CuO/AP microspheres, polyvinylpyrrolidone, energetic materials, mechanical properties

## Abstract

Al-based nanocomposite energetic materials have broad application prospects in explosives and propellants, owing to their excellent energy release efficiency. However, their insufficient reliability, poor stability, and difficulty of formation limit their practical application. This study employed self-assembly using a hydrophilic polymer polyvinylpyrrolidone (PVP) together with nano-aluminum powder (Al), copper oxide (CuO), and ammonium perchlorate (AP) to obtain high-strength and high-activity composite micrometer-sized microspheres. The influence of PVP concentration on the mechanical behavior of Al/AP composite microspheres was systematically investigated, and Al was replaced with ultrasonically dispersed Al/CuO to explore the mechanism of action of PVP in the system and the catalytic behavior of CuO. PVP significantly enhanced the interfacial bonding strength. The Al/AP/5%PVP microspheres achieved a strength of 8.4 MPa under 40% compressive strain, representing a 365% increase relative to Al/AP. The Al/CuO/AP/5%PVP microspheres achieved a strength of 10.2 MPa, representing a 309% increase relative to Al/CuO. The mechanical properties of the composite microspheres were improved by more than threefold, and their thermal reactivities were also higher. This study provides a new method for the controlled preparation of high-strength, high-activity, micrometer-sized energetic microspheres. These materials are expected to be applied in composite solid propellants to enhance their combustion efficiency.

## 1. Introduction

Aluminum (Al) is a high-energy fuel commonly used in composite solid propellants [1,2,3] that can significantly increase the theoretical specific impulse of the propellant [4]. However, the Al_2_O_3_ passivation layer on the surface of micron-sized Al particles has a high boiling point (approximately 3000 K), leading to agglomeration of Al during propellant combustion [5], which reduces its combustion efficiency. To address this issue, researchers have developed various methods to enhance the oxidative activity of Al, such as coating Al surfaces with other materials [6], forming aluminothermic agents with metal oxides, and reducing Al particle size to the nanoscale [7]. Among these, the use of nano-Al particles offers advantages such as high surface energy, low ignition temperature, and high reactivity. The incorporation of nano-Al into propellants can significantly improve Al combustion efficiency [8], reduce agglomeration [9] and ignition delay [10], increase thermal feedback to the combustion surface [11], and enhance the propellant burn rate [12], leading to intense interest in the use of nano-AI in propellants. However, nano-Al has a large specific surface area, and its introduction into propellants can cause excessive paste viscosity [13], making casting difficult. Therefore, the micronizing of nano-Al powder is an important research direction.

Self-assembled nanoenergetic materials (NEMs) have been developed by exploiting and tailoring the interactions between fuels and oxidizers. The reported approaches include (i) electrostatic assembly, (ii) DNA-mediated self-assembly, (iii) polymer-mediated self-assembly, and (iv) functionalized graphene-directed self-assembly [14]. These self-assembled NEMs exhibited combustion properties that are enhanced by several orders of magnitude compared with those of randomly mixed composites. To enable micron-sized aluminum (Al) powder to exhibit nanoscale oxidative activity, Al has been combined with common propellant components to form nanoscale composite energetic materials. In composite solid propellants for which ammonium perchlorate (AP) is a widely used oxidizer, mixtures of AP and Al constitute 60–90% of the total composition [15]. The morphology, relative content, distribution, and other physicochemical properties of these components significantly influence propellant combustion performance. Furthermore, copper oxide (CuO), a common combustion catalyst used in propellants, promotes AP decomposition. The nanoscale Al/CuO composite system has been shown to substantially enhance both Al reactivity and heat release [16]. Consequently, various techniques have been widely adopted to obtain Al/CuO, CuO/AP, and Al/CuO/AP composite systems. Al/CuO is typically synthesized via sol–gel [17], self-assembly [18], ultrasonic dispersion [19], or electrospray [20] methods to achieve intimate nanoscale contact, CuO/AP is prepared using the solvent/non-solvent method [21] to load CuO onto AP crystals, and granulation coating [22] or vapor-induced assembly [23] are employed for the Al/CuO/AP ternary system to synergistically enhance the combustion performance of Al/AP and the catalytic efficiency of CuO. The primary goal of these synthesis methods is to achieve nanoscale component sizes, uniform dispersion, and efficient interfacial contact between the components. Unfortunately, conventional methods, such as ultrasonic dispersion, self-assembly, sol–gel, and drowning-out/agglomeration (D/A) [24], often yield nonspherical composites or low mechanical strength. Moreover, the morphology formed during mixing may not be stable under complex environmental conditions [25], compromising the reliability and stability of the composite. Excellent mechanical properties are crucial for the safety, stability, and effectiveness of energetic materials for practical applications. Stronger materials exhibit superior resistance to damage from impact, friction, and thermal stimulation, thereby significantly mitigating the risk of accidental explosions and ensuring reliable performance. Therefore, the development of innovative strategies for the preparation of high-strength, large-particle composite microspheres is highly significant.

Polyvinylpyrrolidone (PVP) is a nontoxic, nonionic, and hydrophilic polymer. The amide groups on the pyrrolidone rings endow PVP with excellent water solubility, enabling it to act as a medium for regulating particle assembly in aqueous systems [26,27]. Moreover, polar functional groups such as the C=O and C-N groups in the PVP molecule play a key role in regulating particle self-assembly. Specifically, the C=O group can form hydrogen bonds with hydroxyl groups (–OH) on metal surfaces and with the ammonium groups in ammonium perchlorate (AP). Additionally, the C-N group can form hydrogen bonds with the hydroxyl groups on metal surfaces. Furthermore, the electron lone pairs in the C=O and C-N groups can form coordinate bonds with metal atoms. These strong intermolecular forces enable effective assembly of various particles into composite materials. Furthermore, the strong interparticle interactions between PVP and the particles enhance the wetting of the particles by PVP, enabling a large bonding area between the particles and polymers, and even resulting in a coating effect. This allows PVP to function both as a coating layer to inhibit the slow oxidation of nanomaterials and as a matrix for composite materials to enhance their mechanical properties. Based on the above-described advantages, this work selected PVP as the primary medium, combined with capillary forces formed in the aqueous system to prepare the designed hundred-micrometer-scale composite energetic material [28,29,30,31] and conduct an in-depth investigation into the mechanism of action of PVP and the catalytic effect of CuO. The developed PVP-regulated self-assembly method provides new insights into self-assembly and performance optimization of high-energy materials.

## 2. Materials and Methods

### 2.1. Materials

Nano-aluminum powder (Al): particle size 70 nm, purity 99.9% (obtained from Xuzhou Jie Innovative Material Technology Co., Ltd., Xuzhou, China); nano-CuO: particle size 30 nm (obtained from Shanxi Xing’an Chemical Plant in Taiyuan, China); ammonium perchlorate (AP, NH_4_ClO_4_): particle size 125–180 μm: purity 99.5% (obtained from Shanxi Xing’an Chemical Plant in Taiyuan, China; deionized water; N-Methylpyrrolidone (NMP): purity 99.9% (obtained from Shanghai Aladdin Bio-Chemical Technology Co., Ltd., Shanghai, China) as a solvent; Dichloromethane (DCM): purity 99.5% (obtained from Shanghai Aladdin Bio-Chemical Technology Co., Ltd., Shanghai, China) as an anti-solvent; Polyvinylpyrrolidone (PVP, K15): obtained from Shanghai Aladdin Bio-Chemical Technology Co., Ltd., China.

### 2.2. Preparation Method

#### 2.2.1. Preparation of Al/AP/PVP Samples

The following process was used for the preparation of the composite microspheres by PVP-controlled self-assembly. First, 70 nm Al (0.3 g) and DCM (500 g) were added to a 1000 mL three-neck flask and were mixed uniformly by mechanical stirring (200 rpm). Then, a solution composed of AP (6 g) and NMP (18 g) was added dropwise using a constant-pressure dropping funnel. During this process, an ultrasonic dispersion instrument (40 kHz, 400 W) was used to carry out ultrasonication for 10 min. After ultrasonication, the stirring speed was increased to 300 rpm, and an aqueous solution of PVP (3 g) was added dropwise over 30 min. After the addition was completed, stirring was continued for 10 min, and then the solution was filtered under vacuum and dried at room temperature under vacuum for 12 h to obtain Al/AP/x% PVP (where x% represents the concentration of PVP) composite microspheres. Throughout the process, the solution temperature was maintained at approximately 5 °C using a cooling circulation system. Additionally, Al/AP microspheres without PVP were prepared as blank samples. The parameters of the prepared samples (a–c) and (d) are shown in Table 1.

#### 2.2.2. Preparation of Al/CuO/AP/PVP

First, an Al/CuO composite system with an equivalent ratio Φ of 1.2 was prepared using the ultrasonic dispersion method to replace Al. The original process was used to obtain Al/CuO/AP/x%PVP composite microspheres (where x% represents the concentration of PVP). Al/CuO/AP microspheres without PVP were used as blank samples. The parameters of the prepared samples (e) and (f) are listed in Table 1. The equivalent ratio Φ represents the fuel/oxidizer ratio [32].

$$\phi =\frac{{\left(m_{F}∕m_{o}\right)}_{a}}{{\left(m_{F}∕m_{o}\right)}_{b}}$$

where (m_F_/m_O_)_a_ ${\left(m_{F}∕m_{o}\right)}_{a}$ is the actual mass ratio of fuel to oxidizer, and ${\left(m_{F}∕m_{o}\right)}_{b}$ is the mass ratio of fuel to oxidizer at stoichiometric equilibrium.

**Figure 1:**
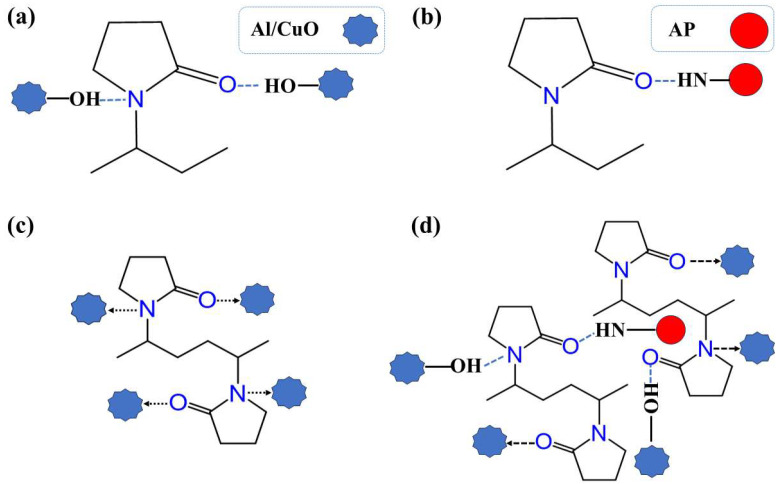
(**a**) Hydrogen bonds in Al/CuO–PVP, (**b**) hydrogen bonds in AP–PVP, (**c**) coordination bonds in Al/CuO–PVP, and (**d**) bonds formed by PVP between Al, CuO, and AP.

Figure 1 illustrates the forces between PVP and various other components during self-assembly, including hydrogen and coordination bonds.

### 2.3. Fourier-Transform Infrared Spectroscopy (FT-IR) Characterization of Molecular Groups

The infrared spectra of the composite particles were measured using a Fourier-transform infrared spectrometer (FT-IR, Bruker Tensor27, Berlin, Germany) and the accompanying 2012 version of OPUS software. Prior to testing, the samples were dried, ground with KBr at a ratio of 1:100, and pressed for 30 s at 10 MPa to prepare the samples. Testing was conducted in the range of 400–4000 cm^−1^ with a resolution of 4 cm^−1^ and 32 cumulative scans.

### 2.4. X-Ray Diffraction

The samples were analyzed using a Bruker D8A instrument. X-ray diffraction (XRD) patterns were recorded in the range of 10–90°, using Cu-Kα radiation (λ = 1.5406 Å) at 40 mA and 25 kV, with a Bragg–Brentano geometry and a scan step of 0.01°. The exposure time for each point was 1.67 s, with no sample rotation.

### 2.5. Scanning Electron Microscopy

The surface morphologies and elemental distributions of the samples were observed using a field-emission scanning electron microscope (FE-SEM, MERLIN Compact, Oberkochen, Germany), and SEM micrographs of the samples were obtained.

### 2.6. Mechanical Property Testing

The samples were tested using an INSTRON instrument (825 University Ave, Norwood, MA, USA). The original samples were sieved using standard sieves to ensure consistent particle size, with each compression test using 0.15 g of sample material. The sample particles were compressed at a rate of 5 mm/min using a universal testing machine.

### 2.7. Thermal Decomposition Performance Testing

The tests were conducted using equipment from METTLER TOLEDO (Madde, Switzerland). For each sample, the mass was less than 1 mg. All tests were conducted a heating rate of 10 °C/min, with nitrogen atmosphere, and the heating temperature ranged from room temperature to 500 °C.

## 3. Results

### 3.1. FTIR Analysis

**Figure 2:**
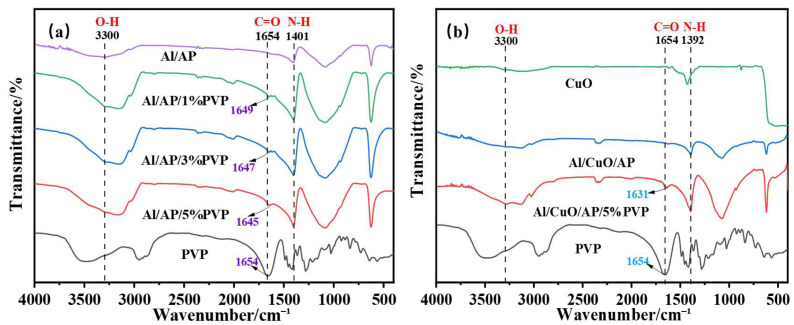
(**a**) Al/AP, Al/AP/1%PVP, Al/AP/3%PVP, Al/AP/5%PVP, PVP, and (**b**) CuO, Al/CuO/AP, Al/CuO/AP/PVP, PVP have distinct specific vibration bands (expressed in cm^−1^) in their Fourier transform infrared spectra (FTIR).

Figure 2a shows the FTIR spectra of Al/AP, Al/AP/1%PVP, Al/AP/3%PVP, Al/AP/5%PVP, and pure PVP. In the Al/AP system, the following characteristic peaks were observed: the broad peak at 3300 cm^−1^ corresponds to the superposition of O-H and N-H stretching vibrations [33,34], the peak at 1401 cm^−1^ corresponds to N-H bending vibrations, and the peaks at 1082 cm^−1^ and 624 cm^−1^ can be attributed to the vibrations of ClO_4_^−^ ions [35]. Upon the introduction of PVP into the Al/AP system, the positions of the characteristic peaks shifted. Compared with the C=O stretching vibration peak (1654 cm^−1^) in pure PVP, the redshift of this peak in the PVP-added sample indicates the presence of a chemical interaction between the C=O group and the metal atom, such as the formation of a coordination bond. Furthermore, as the PVP concentration increased, the redshift also increased, indicating that the strength of the coordination bonds increases with higher PVP concentration [36]. Additionally, the N-H bending vibration peak at 1401 cm^−1^ also shifts to lower wavenumbers, while the absorption peak near 3300 cm^−1^ broadens, collectively indicating the formation of hydrogen bonds [37]. These phenomena indicate that PVP enhances the strength of interfacial bonding by forming a coordination bond/hydrogen bond network between Al and PVP-AP.

Figure 2b shows the FT-IR spectra of CuO, Al/CuO/AP, Al/CuO/AP/5%PVP, and pure PVP. The spectra show characteristics that are generally similar to those of the spectra presented in Figure 2a; however, some peaks are shifted. Notably, the C=O stretching vibration peak of Al/CuO/AP/5%PVP shows a redshift of up to 23 cm^−1^ compared to pure PVP (1654 cm^−1^), indicating that the introduction of CuO significantly enhances the coordination interaction between PVP carbonyl groups and the metal [38]. Additionally, the shifts in other functional groups (such as N-H) and the significant broadening of the absorption peak near 3300 cm^−1^ further confirm that CuO promotes the enhancement of the hydrogen bonding network within the system [39].

Comparison of Figure 2a with Figure 2b shows the following: (1) After adding CuO, the redshift of the C=O stretching vibration peak (from the reference value of 1654 cm^−1^) in the Al/CuO/AP/PVP system (e.g., 23 cm^−1^ for Al/CuO/AP/5%PVP) is significantly greater than that in the Al/AP/PVP system (e.g., 9 cm^−1^ for Al/AP/5%PVP). (2) The absorption peak near 3300 cm^−1^ (assigned to the O-H/N-H stretching vibrations) in Figure 2b exhibits a significantly broader peak compared to the corresponding sample in Figure 2a.

### 3.2. XRD Analysis

**Figure 3:**
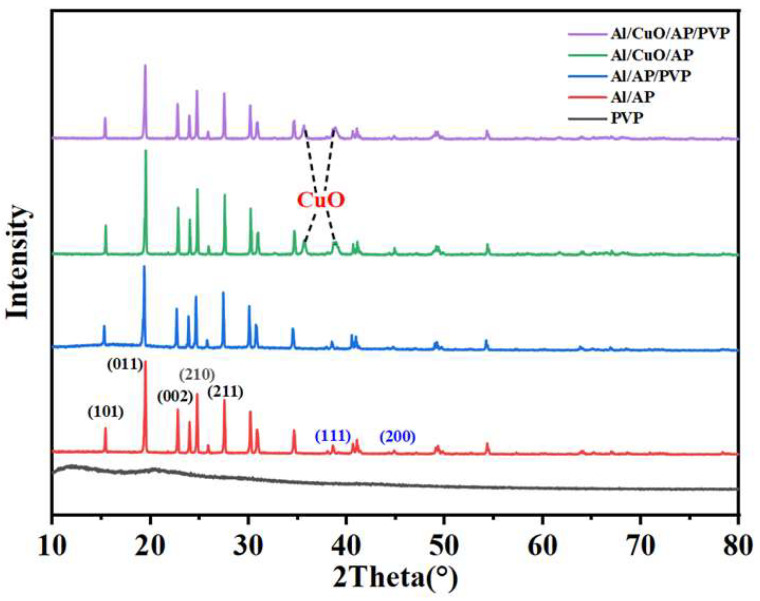
X-ray diffraction (XRD) patterns of PVP, Al/AP, Al/AP/PVP, Al/CuO/AP, and Al/CuO/AP/PVP.

Figure 3 shows the XRD patterns of Al/AP, Al/AP/PVP, Al/CuO/AP, and Al/CuO/AP/PVP. The peaks at 15.3°, 19.4°, 23.8°, 24.7°, and 27.5° correspond to the (101), (011), (002), (210), and (211) crystal planes of AP (AP PDF43-0648), while the peaks at 38.5°, 44.7°, 65.2°, and 78.3° correspond to the (111), (200), (220), and (311) crystal planes of Al (Al PDF-0787). This confirms the successful preparation of Al/AP samples via the developed PVP-regulated self-assembly method. The characteristic peaks of CuO appeared at 35.57° and 38.74°, further confirming the successful introduction of CuO into the Al/AP system. The introduction of PVP did not alter the crystalline phases of the components, which is consistent with the conclusions of the aforementioned analysis.

After adding PVP, the XRD pattern did not show significant changes for the following two reasons: (1) PVP does not react chemically with Al/AP but is merely physically mixed; therefore, its main characteristic peaks do not undergo significant changes, and (2) PVP is typically an amorphous polymer material. Amorphous materials give rise to broad diffuse peaks in the XRD spectrum. Natural PVP is inherently amorphous with weak characteristic features, and thus does not show prominent peaks in the XRD spectrum [40].

### 3.3. SEM Morphological Analysis and Macro Sample Analysis

**Figure 4:**
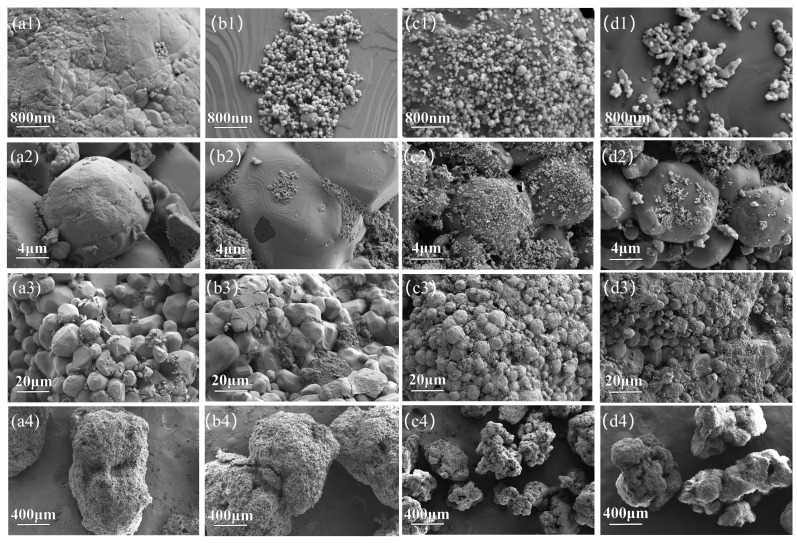
Scanning electron microscopy (SEM) micrographs of (**a1**–**a4**) Al/AP, (**b1**–**b4**) Al/AP/5%PVP, (**c1**–**c4**) Al/CuO/AP, and (**d1**–**d4**) Al/CuO/AP/5%PVP.

Figure 4 shows SEM images of the Al/AP, Al/AP/5%PVP, Al/CuO/AP, and Al/CuO/AP/5%PVP composite microspheres. For the Al/AP composite microspheres (Figure 4(a1–a4)), numerous defects are visible on the surface at the microscopic scale (Figure 4(a1)). Higher-magnification images (Figure 4(a2)) reveal extensive gaps between AP particles, and an examination of a wider field of view (Figure 4(a3)) further confirms the presence of significant interparticle voids. Figure 4(a4) illustrates the morphology of the microspheres formed by the assembly of Al and AP. In contrast to the Al/AP microspheres, the Al/AP/5%PVP composite microspheres (Figure 4(b1–b4)) exhibit a significantly improved microstructure. Figure 4(b1) shows that the surface of the AP particles was wavy and smoother, with nanosized Al particles tightly adhering to the AP particles’ surfaces, indicating that PVP successfully coated the AP and promoted strong interfacial bonding with Al. Compared to the rough AP surface and evident interparticle gaps in the system without PVP (Figure 4(a2)), smoother AP particle surfaces and a significant reduction in interparticle gaps were observed due to the addition of PVP (Figure 4(b2)), demonstrating that PVP effectively filled the gaps between the AP particles and enhanced interfacial bonding. Figure 4(b4) shows the corresponding microspherical morphologies.

Compared to the Al/AP system containing only nano-Al (Figure 4(a1)), the number of nanoparticles attached to the AP surface increased significantly (Figure 4(c1)) for the Al/CuO/AP composite microspheres (Figure 4(c1–c4)), confirming the introduction of nano-CuO. However, the microsphere surface still exhibited certain defects, and the particle were still relatively dispersed (Figure 4(c1)). The microspheres formed by this assembly exhibited irregular spherical shapes (Figure 4(c4)). The Al/CuO/AP/5%PVP composite microspheres formed after adding PVP (Figure 4(d1–d4)) show significant changes; the AP surface becomes smoother, and the Al/CuO nanoparticles form more compact agglomerates (Figure 4(d1)), indicating that PVP not only coats the AP but also forms a tight connection with the Al/CuO particles. A comparison of Figure 4(c2,d2) further confirms the role of PVP in filling the gaps between the AP particles and shows that the microsphere morphology of Al/CuO/AP/5%PVP (Figure 4(d4)) is more regular than that of Al/CuO/AP without PVP (Figure 4(c4)).

High-magnification images (Figure 4(a3–d3)) further support the above conclusions, as it is observed that the AP particle surfaces in the PVP-added samples (Figure 4(b3,d3)) were significantly smoother than those in the untreated samples (Figure 4(a3,c3)), with a marked reduction in interparticle gaps. Additionally, as shown in Figure 4(a4–d4), the particle sizes of the composite microspheres are distributed within the range of 400–800 μm, confirming that the self-assembly strategy successfully achieved the preparation of micron-sized microspheres.

**Figure 5:**
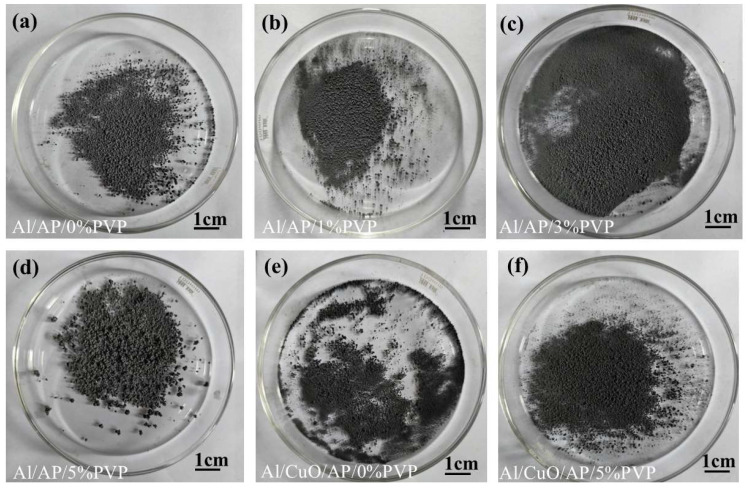
(**a**) Al/AP/0%PVP. (**b**) Al/AP/1%PVP. (**c**) Al/AP/3%PVP. (**d**) Al/AP/5%PVP. (**e**) Al/CuO/AP/0%PVP. (**f**) Al/CuO/AP/5%PVP.

Figure 5 shows the morphological characteristics of the Al/AP/0%PVP, Al/AP/1%PVP, Al/AP/3%PVP, Al/AP/5%PVP, Al/CuO/AP, and Al/CuO/AP/5%PVP composite microspheres.

**Figure 6:**
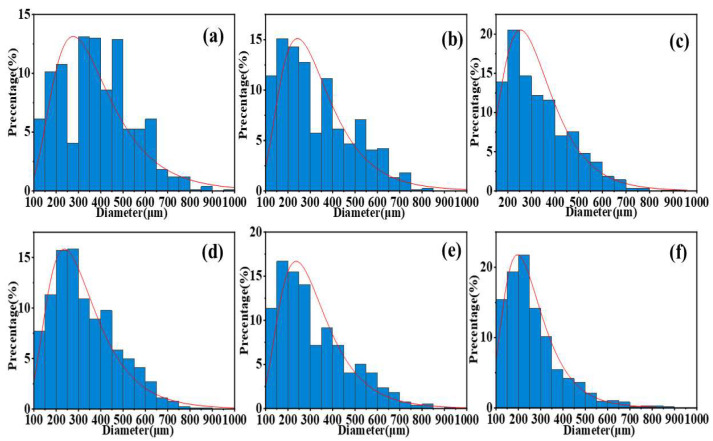
Size distribution of the sample. (**a**) Al/AP/0%PVP. (**b**) Al/AP/1%PVP. (**c**) Al/AP/3%PVP (**d**) Al/AP/5%PVP. (**e**) Al/CuO/AP/0%PVP. (**f**) Al/CuO/AP/5%PVP.

Comparison of Figure 6a–d shows that as the PVP content increases, the variance of the particle size distribution of the samples decreases significantly, and the particle size distribution gradually concentrates in the 200–300 μm range. This indicates that the size distribution of the particles prepared using our method tends to be uniform, and its distribution pattern is closer to a normal distribution curve. This conclusion is further corroborated by the comparison of Figure 6e,f. These results clearly demonstrate the significant role of PVP in improving the uniformity of the particle size distribution of the sample.

Notably, as the PVP content increased from 0 to 5%, the surface morphology of the microspheres showed a clear improvement trend, transitioning from an initial rough, porous structure to a smooth and dense spherical structure. The Al/CuO/AP microspheres maintained a good sphericity in the composite system containing CuO, whereas the addition of 5% PVP (Al/CuO/AP/5% PVP) resulted in smoother microsphere surfaces and more compact particle packing. These changes in the morphological characteristics were closely related to the coating effect and interfacial modification of PVP, further confirming the important role of PVP in improving the structural uniformity and surface properties of composite microspheres [41,42,43].

### 3.4. Mechanism Analysis

**Figure 7:**
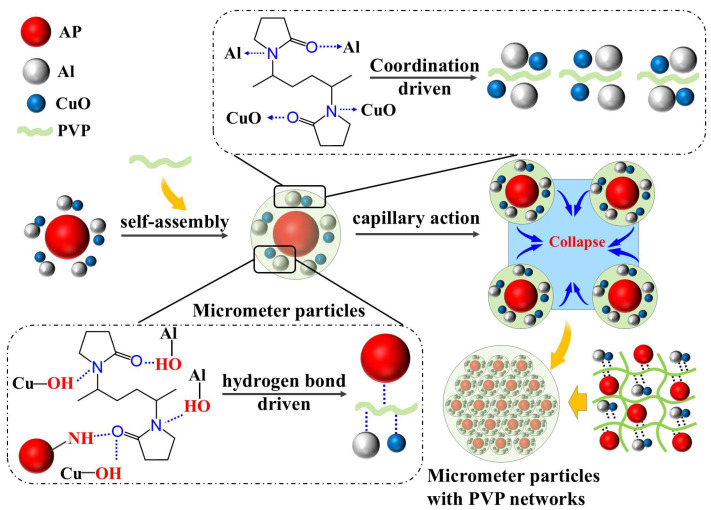
Schematic diagram of the self-assembly of Al/CuO/AP/PVP composite microspheres regulated by polyvinylpyrrolidone (PVP).

This study employed an innovative self-assembly regulation strategy to achieve the controlled preparation of Al/CuO/AP/PVP composite microspheres through multiscale synergistic effects. The self-assembly process is illustrated in Figure 7. Nano-sized Al/CuO particles were first premixed with micron-sized AP particles to form a nano/micron composite mixture. Subsequently, a solution of the hydrophilic polymer PVP was introduced as a framework. Their unique amphiphilic properties (hydrophilic pyrrolidone rings and hydrophobic carbon chains) allow for the formation of a molecular bridging network in the aqueous phase. During self-assembly, PVP enhances the interface through multiple mechanisms; the C=O/C-N groups of PVP form hydrogen bonds with the hydroxyl groups (Al-OH and Cu-OH) on the Al/CuO surface, and the C=O group can form hydrogen bonds with the N-H groups of AP, while the electron lone pairs of PVP form coordination bonds with Al and CuO [36,38], constructing a three-dimensional cross-linked network. During the wetting of the particles by the aqueous solution, strong capillary forces were generated, which, together with hydrogen bond-driven forces, promoted close aggregation of the particles, thereby achieving the synergistic assembly of the Al/CuO/AP/PVP composite microspheres.

This self-assembly strategy establishes a synergistic mechanism involving hydrogen bonding, coordination bond assembly, and solvent bridging, which enhances the mechanical properties of Al/CuO/AP. Here, hydrogen bond/coordination bond assembly refers to the formation of bonds between particles through coordination bonds/hydrogen bonds, thereby creating microparticles, and solvent bridging refers to the PVP aqueous solution utilizing capillary forces to cause the aggregated microparticles to collide and polymerize, forming larger composite microspheres. Additionally, by adjusting the PVP concentration (1%, 3%, and 5%), the bonding strength can be controlled to prepare composite microspheres with varying strengths.

### 3.5. Mechanical Properties of Composite Particles at Different PVP Concentrations

**Figure 8:**
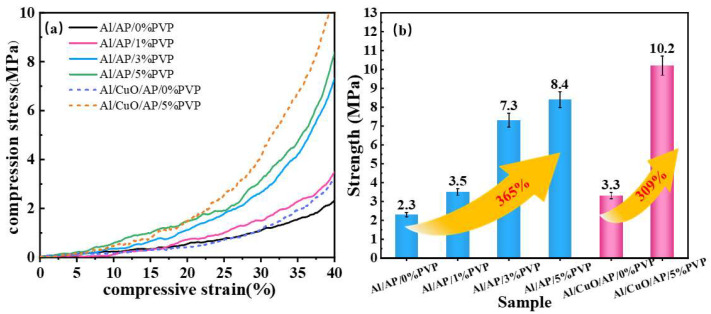
(**a**) Stress–average strain graphs for the selected specimens and (**b**) strength at 40% compressive strain.

Using a universal testing machine, composite microspheres with uniform mass and size, including Al/AP, Al/AP/1%PVP, Al/AP/3%PVP, Al/AP/5%PVP, Al/CuO/AP, and Al/CuO/AP/5%PVP, were compressed in an impact sensitivity mold until the compression strain reached 40%. The obtained stress–strain curves are shown in Figure 8a and indicate a significant positive correlation between the composite microsphere strength and the PVP concentration. Figure 8b quantitatively demonstrates that the strength ranking of the Al/AP system is Al/AP < Al/AP/1%PVP < Al/AP/3%PVP < Al/AP/5%PVP, with the addition of 5% PVP resulting in the strength of 8.4 MPa, corresponding to an increase in the strength by 365% relative to that of Al/AP. The increased slope of the stress–strain curve further confirms the significant enhancement of material strength. For the CuO-containing systems, the strength ranking is Al/CuO/AP < Al/CuO/AP/5%PVP, with the addition of 5% PVP resulting in the strength of 10.2 MPa, which corresponds to a 309% increase relative to that of Al/CuO/AP, validating the universality of PVP interface enhancement. The high strength enhancement primarily stems from the crosslinked network formed by PVP, which serves as a structural framework, with its C=O/C-N groups forming hydrogen bonds/coordination bonds with Al/AP to create a crosslinked network. This network enables the material to absorb a large amount of energy under stress and inhibits crack propagation, endowing the material with exceptional damage tolerance properties. In the Al/CuO/AP ternary system, PVP achieved high strength by bridging the Al/CuO-AP multi-level interfaces. Both macroscopic mechanical behavior and microscopic structural analyses confirmed the importance and effectiveness of the PVP addition.

### 3.6. TG/DTG Thermal Decomposition

**Figure 9:**
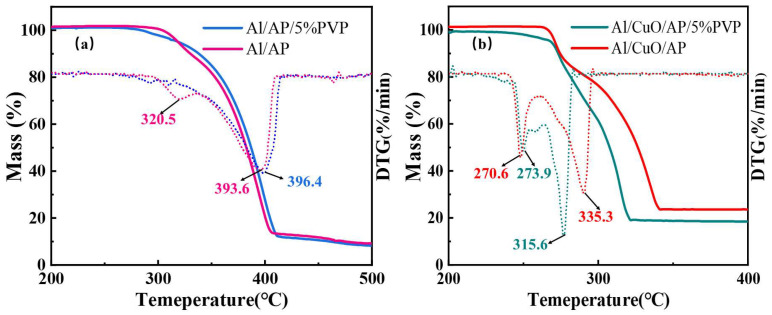
TG and DTG curves of (**a**) Al/AP, Al/AP/5%PVP and (**b**) Al/CuO/AP, Al/CuO/AP/5%PVP.

Figure 9 shows the results of the TG thermal decomposition tests conducted on Al/AP, Al/AP/5%PVP, Al/CuO/AP, and Al/CuO/AP/5%PVP, together with the DTG curves obtained by differentiation of the TG curves. For each sample, the low-temperature decomposition temperature (LTD) and high-temperature decomposition temperature (HTD), as determined from the TG/DTG plots, are presented in Table 2. HTD is the most important parameter for describing the decomposition behavior of AP [44] because a lower HTD can reduce the ignition delay time and increase the combustion rate of the propellant [45,46]. Additionally, improving the thermal release efficiency of AP decomposition can enhance the specific impulse (Isp) [47,48].

As shown in Table 2 and Figure 9a, the exothermic decomposition temperature (HTD) of the Al/AP composite material is lower than that of pure AP [49], indicating that Al promotes AP decomposition. Furthermore, the addition of PVP slightly increases the HTD of the Al/AP composite material, and only a single decomposition peak is observed on its TG curve, which is attributed to PVP coating the AP, which causes the HTD and LTD to merge [50,51]. However, in the Al/CuO/AP system (Figure 9b), the HTD of AP was significantly reduced by approximately 60 °C, demonstrating that CuO has a strong catalytic effect on the decomposition of AP, significantly enhancing the reactivity of the composite energetic microspheres. Notably, the HTD values of the samples with added PVP were even lower, indicating that PVP influenced this system, further enhancing the catalytic activity of CuO toward AP at high temperatures [52].

## 4. Discussion

In summary, as demonstrated in the article, PVP plays several roles in the composites, listed as follows:

(1) PVP acts as a framework, forming coordination and hydrogen bonds with metals and AP, strengthening the intermolecular interactions, and improving the mechanical properties of the entire system.
(2) While enhancing the mechanical properties, the PVP solution promoted the formation of composite microspheres through the combined effects of hydrogen-bond-driven, coordination-bond-driven, and capillary forces.
(3) In the Al/CuO/AP/PVP system, PVP interacted with CuO to enhance its catalytic performance.

## 5. Conclusions

In this study, PVP was used as a medium to combine self-assembly with capillary forces to achieve the ordered growth of particles. This process results in the preparation of composite microspheres with macroscopic dimensions reaching hundreds of micrometers and microscopic structures at the nanoscale, characterized by uniform mixing. During this process, the C=O groups in the PVP molecules and the C-N groups on the pyrrolidone rings can form hydrogen bonds and coordination bonds with aluminum powder and CuO, respectively. The C=O group forms hydrogen bonds with the N-H groups on the AP surface. These interactions enable the nanoparticles and AP to form uniformly dispersed micrometer-sized particles, which aggregate into composite microspheres with sizes of hundreds of micrometers under the capillary forces of the PVP solution. Because PVP exhibits strong intermolecular interactions with all three components in the microspheres, the resulting composite microspheres exhibit excellent wetting by PVP of the particles within the microspheres, creating a coating-like effect. Thus, PVP serves as a mechanical skeleton in the composite microspheres, significantly enhancing their compressive strength. In addition, because the particles within the composite microspheres aggregate through self-assembly, the components are uniformly mixed. CuO exhibits a significant catalytic effect on the decomposition of AP, resulting in composite microspheres with high reactivity. The high strength of the composite microspheres is expected to maintain their morphological structure during the propellant slurry mixing process, whereas their larger size ensures smooth casting of the propellant slurry. High reactivity is expected to enhance the combustion efficiency of propellants. Therefore, the hundred-micron-sized energetic composite microspheres prepared in this study have potential applications as composite solid propellants. Future research will focus on exploring their impact on the propellant manufacturing processes and combustion performance.

## Figures and Tables

**Table 1:** The composition of the substance used in the text.

Sample	Polymer Matrix	x (%)
(a) Al/AP	-	*-*
(b) Al/AP	PVP	1%
(c) Al/AP	PVP	3%
(d) Al/AP	PVP	5%
(e) Al/CuO/AP	-	-
(f) Al/CuO/AP	PVP	5%

**Table 2:** The peak temperature from DTG of different samples.

Samples	Stage	(Tm/°C)
Al/AP	LTD	320.5
	HTD	393.6
Al/AP/5%PVP	HTD	396.4
Al/CuO/AP	LTD	270.6
	HTD	335.3
Al/CuO/AP/5%PVP	LTD	273.9
	HTD	315.6

## Data Availability

The original contributions presented in this study are included in the article. Further inquiries can be directed to the corresponding authors.

## Abbreviations

The following abbreviations are used in this manuscript:

PVP	Polyvinylpyrrolidone
Al	Aluminum
CuO	Copper oxide
AP	Ammonium perchlorate
NEMs	Nanoenergetic materials
NMP	N-Methylpyrrolidone
DCM	Dichloromethane
LTD	Low-temperature decomposition temperature
HTD	High-temperature decomposition temperature

## References

[1] Pang W., Fan X., Wang K., Chao Y., Xu H., Qin Z., Zhao F. (2020). Al-Based Nano-Sized Composite Energetic Materials (Nano-CEMs): Preparation, Characterization, and Performance. Nanomaterials.

[2] Liu S., Zhang C., Wang Y., Wei X. (2025). Synthesis of Energetic Materials by Microfluidics. Def. Technol..

[3] Wang W., Li H., Zhang M., Zhao F., Xu S., Wang C., Qin Z., An T., Xu K. (2022). Effects of Oxidizer and Architecture on the Thermochemical Reactivity, Laser Ignition and Combustion Properties of Nanothermite. Fuel.

[4] Sippel T.R., Son S.F., Groven L.J. (2014). Aluminum Agglomeration Reduction in a Composite Propellant Using Tailored Al/PTFE Particles. Combust. Flame.

[5] Sippel T.R., Son S.F., Groven L.J., Zhang S., Dreizin E.L. (2015). Exploring Mechanisms for Agglomerate Reduction in Composite Solid Propellants with Polyethylene Inclusion Modified Aluminum. Combust. Flame.

[6] Campbell L.L., Hill K.J., Smith D.K., Pantoya M.L. (2021). Thermal Analysis of Microscale Aluminum Particles Coated with Perfluorotetradecanoic (PFTD) Acid. J. Therm. Anal. Calorim..

[7] Comet M., Martin C., Schnell F., Spitzer D. (2017). Nanothermite Foams: From Nanopowder to Object. Chem. Eng. J..

[8] Dreizin E.L. (2009). Metal-Based Reactive Nanomaterials. Prog. Energy Combust. Sci..

[9] Jayaraman K., Chakravarthy S.R., Sarathi R. (2011). Quench Collection of Nano-Aluminium Agglomerates from Combustion of Sandwiches and Propellants. Proc. Combust. Inst..

[10] Arkhipov V.A., Korotkikh A.G. (2012). The Influence of Aluminum Powder Dispersity on Composite Solid Propellants Ignitability by Laser Radiation. Combust. Flame.

[11] Dokhan A., Price E.W., Seitzman J.M., Sigman R.K. (2002). The Effects of Bimodal Aluminum with Ultrafine Aluminum on the Burning Rates of Solid Propellants. Proc. Combust. Inst..

[12] Jacob R.J., Jian G., Guerieri P.M., Zachariah M.R. (2015). Energy Release Pathways in Nanothermites Follow through the Condensed State. Combust. Flame.

[13] Bondarenko A., Islamov S., Ignatyev K., Mardashov D., Saint Petersburg Mining University (2020). Laboratory Studies of Polymer Compositions for Well-Kill under Increased Fracturing. Perm J. Pet. Min. Eng..

[14] Thiruvengadathan R., Wang A. (2024). Nanoenergetic Materials: From Materials to Applications. Nanomaterials.

[15] Muthiah R., Krishnamurthy V.N., Gupta B.R. (1992). Rheology of HTPB Propellant. I. Effect of Solid Loading, Oxidizer Particle Size, and Aluminum Content. J. Appl. Polym. Sci..

[16] Lyu J.-Y., Xu G., Zhang H., Yang W., Yan Q.-L. (2024). Thermal Decomposition and Combustion Behavior of the Core-Shell Al@AP Composite Embedded with CuO as a Catalyst. Fuel.

[17] Wang Y., Zhang X., Xu J., Shen Y., Wang C., Li F., Zhang Z., Chen J., Ye Y., Shen R. (2021). Fabrication and Characterization of Al–CuO Nanocomposites Prepared by Sol-Gel Method. Def. Technol..

[18] Slocik J.M., Crouse C.A., Spowart J.E., Naik R.R. (2013). Biologically Tunable Reactivity of Energetic Nanomaterials Using Protein Cages. Nano Lett..

[19] Song J., Guo T., Yao M., Chen J., Ding W., Bei F., Mao Y., Yu Z., Huang J., Zhang X. (2020). A Comparative Study of Thermal Kinetics and Combustion Performance of Al/CuO, Al/Fe_2_O_3_ and Al/MnO_2_ Nanothermites. Vacuum.

[20] Wang C., Xu J., Dai J., Wang Y., Shen Y., Zhang Z., Shen R., Ye Y. (2020). Probing the Reaction Mechanism of Al/CuO Nanocomposites Doped with Ammonium Perchlorate. Nanotechnology.

[21] Hosseini S.G., Abazari R. (2015). A Facile One-Step Route for Production of CuO, NiO, and CuO–NiO Nanoparticles and Comparison of Their Catalytic Activity for Ammonium Perchlorate Decomposition. RSC Adv..

[22] Zhou X., Xu R., Nie H., Yan Q., Liu J., Sun Y. (2023). Insight into the Precise Catalytic Mechanism of CuO on the Decomposition and Combustion of Core–Shell Al@AP Particles. Fuel.

[23] Hu Y., Yang S., Tao B., Liu X., Lin K., Yang Y., Fan R., Xia D., Hao D. (2019). Catalytic Decomposition of Ammonium Perchlorate on Hollow Mesoporous CuO Microspheres. Vacuum.

[24] Shim H.-M., Kim J.-K., Kim H.-S., Koo K.-K. (2016). Production of the Spherical Nano-Al/AP Composites by Drowning-Out/Agglomeration and Their Solid-Reaction Kinetics. Ind. Eng. Chem. Res..

[25] Han K., Li S., Tan K., Xie Z., Shi H., Liu Y., An C., Wang J. (2024). In Situ Self-Crosslinking Binder System—Enhances the Mechanical Performance Gain of Composite Energetic Materials. Chem. Eng. J..

[26] Koczkur K.M., Mourdikoudis S., Polavarapu L., Skrabalak S.E. (2015). Polyvinylpyrrolidone (PVP) in Nanoparticle Synthesis. Dalton Trans..

[27] Premalatha M., Vijaya N., Selvasekarapandian S., Selvalakshmi S. (2016). Characterization of Blend Polymer PVA-PVP Complexed with Ammonium Thiocyanate. Ionics.

[28] Zhang J., Wang M., Yao X., Liu J., Yan B. (2024). Thioctic Acid-Based Solvent-Free and Recoverable Adhesive for Dry/Wet Environments. ACS Appl. Mater. Interfaces.

[29] Xing Y., Deng Z., Wang Q., Xiong J., Liu X., Huang L., Zhu Y., Zhang J. (2024). Polymer Lewis Base for Improving the Charge Transfer in Tin–Lead Mixed Perovskite Solar Cells. Nanomaterials.

[30] Pan G., Leng J., Deng L., Xing L., Feng R. (2021). Recording the Self-Assembly Behavior of Nanomaterials Directed by Hydrogen Bonding. Cryst. Growth Des..

[31] Liu Y., Wang L., Zhao L., Zhang Y., Li Z.-T., Huang F. (2024). Multiple Hydrogen Bonding Driven Supramolecular Architectures and Their Biomedical Applications. Chem. Soc. Rev..

[32] Pantoya M.L., Levitas V.I., Granier J.J., Henderson J.B. (2009). Effect of Bulk Density on Reaction Propagation in Nanothermites and Micron Thermites. J. Propuls. Power.

[33] Subashini C., Sivasubramanian R., Sundaram M.M., Priyadharsini N. (2025). The Evolution of Allotropic Forms of Na_2_CoP_2_O_7_ Electrode and Its Role in Future Hybrid Energy Storage. J. Energy Storage.

[34] Dharmalingam S.T., Dar M.A., Gul R., Minakshi Sundaram M., Alnaser I.A., Sivasubramanian R. (2025). Nano-Octahedron Cobalt Oxide Decorated Graphene Nanocomposites for the Selective/Simultaneous Detection of Dopamine. Adv. Mater. Inter..

[35] Zhi J., Tian-Fang W., Shu-Fen L., Feng-Qi Z., Zi-Ru L., Cui-Mei Y., Yang L., Shang-Wen L., Gang-Zhui Z. (2006). Thermal Behavior of Ammonium Perchlorate and Metal Powders of Different Grades. J. Therm. Anal. Calorim..

[36] Gottapu S., Padhi S.K., Krishna M.G., Muralidharan K. (2015). Poly(Vinylpyrrolidone) Stabilized Aluminum Nanoparticles Obtained by the Reaction of SiCl_4_ with LiAlH_4_. New J. Chem..

[37] Zhou L., Tsai H.-W., Kuo T.-W., Kao J.-C., Lo Y.-C., Chang J.-M., Chiang T.-H., Dai S., Wang K.-W., Chen T.-Y. (2025). Atomic Layered ZnO Between Cu Nanoparticles and a PVP Polymer Layer Enable Exceptional Selectivity and Stability in Electrocatalytic CO_2_ Reduction to C_2_H_4_. Adv. Sci..

[38] Khan H.U., Tariq M., Shah M., Iqbal M., Jan M.T. (2020). Inquest of Highly Sensitive, Selective and Stable Ammonia (NH3) Gas Sensor: Structural, Morphological and Gas Sensing Properties of Polyvinylpyrrolidone (PVP)/CuO Nanocomposite. Synth. Met..

[39] Javed R., Ahmed M., Haq I.U., Nisa S., Zia M. (2017). PVP and PEG Doped CuO Nanoparticles Are More Biologically Active: Antibacterial, Antioxidant, Antidiabetic and Cytotoxic Perspective. Mater. Sci. Eng. C.

[40] Khan H.U., Tariq M., Shah M., Jan M.T., Iqbal M., Khan J., Ahsan A.R., Rahim A. (2020). The Efficacy of Polyvinylpyrrolidone (PVP)/CuO Nanocomposite as an Appropriate Room Temperature Humidity Sensing Material: Fabrication of Highly Sensitive Capacitive Resistive Type Humidity Sensor. J. Mater. Sci. Mater. Electron..

[41] Zhou J., Zhou J., Hu Z., Wang L. (2019). Enhancement of Adsorption and Visible Light Photocatalytic Activity of the Zn^2+^-Doped BiOBr/PVP Modified Microspheres for RhB. Mater. Sci. Semicond. Process..

[42] Li J., Jiang Z., Li W. (2020). Preparation and Self-Healing Properties of Clinker/PVP Microsphere in Cement Paste. Materials.

[43] Choi H., Kim H.S. (2023). In Situ Synthesis and Stabilization of PVP-Coated Aluminum Nanoparticles by One-Step Pulsed Laser Ablation in Liquid: Investigation and Quantification of PVP Coverage Effects. Surf. Interfaces.

[44] Zhou L., Cao S., Zhang L., Xiang G., Wang J., Zeng X., Chen J. (2020). Facet Effect of Co_3_O_4_ Nanocatalysts on the Catalytic Decomposition of Ammonium Perchlorate. J. Hazard. Mater..

[45] Zhang Y., Meng C. (2016). Facile Fabrication of Fe_3_O_4_ and Co_3_O_4_ Microspheres and Their Influence on the Thermal Decomposition of Ammonium Perchlorate. J. Alloys Compd..

[46] Cao S., Han X., Zhang L., Wang J., Luo Y., Zou H., Chen J. (2019). Facile and Scalable Preparation of α-Fe_2_O_3_ Nanoparticle by High-Gravity Reactive Precipitation Method for Catalysis of Solid Propellants Combustion. Powder Technol..

[47] Hao G., Liu J., Liu H., Xiao L., Qiao Y., Gao H., Jiang W., Zhao F. (2016). Cu–Cr–Pb Nanocomposites: Synthesis, Characterization and Their Catalytic Effect on Thermal Decomposition of Ammonium Perchlorate. J. Therm. Anal. Calorim..

[48] Boldyrev V.V. (2006). Thermal Decomposition of Ammonium Perchlorate. Thermochim. Acta.

[49] Ma G., Ma Z., Zhang Z., Yang Z., Lei Z. (2012). Synthesis and Catalytic Properties of Mesoporous Alumina Supported Aluminium Chloride with Controllable Morphology, Structure and Component. J. Porous Mater..

[50] Liu W., Xie Y., Xie Q., Fang K., Zhang X., Chen H. (2019). In Situ Synthesis of Hydrophobic Coatings: An Effective Strategy to Reduce Hygroscopicity and Catalyze Decomposition of Ammonium Perchlorate. CrystEngComm.

[51] Huang Y., Tian X., Wang J., Zhong K., Chen Y., Li C., Jia P. (2024). Bioinspired Fabrication of an Insensitive Ammonium Perchlorate Core–Shell Composite with Polydopamine Coating. Polymers.

[52] Shahmiri M., Bayat S., Kharrazi S. (2023). Catalytic Performance of PVP-Coated CuO Nanosheets under Environmentally Friendly Conditions. RSC Adv..

